# Life Course Stressors, Latent Coping Strategies, Alcohol Use, and Adherence among People with HIV

**DOI:** 10.1007/s10461-024-04541-6

**Published:** 2024-11-15

**Authors:** Amrita Gill, Gretchen Clum, Patricia Molina, David Welsh, Tekeda Ferguson, Katherine P. Theall

**Affiliations:** 1 Department of Psychiatry and Human Behavior, Warren Alpert Medical School of Brown University, Box G-BH, Providence, Rhode Island, RI 02912 USA; 2 Department of Social, Behavioral and Population Sciences, Celia Scott Weatherhead School of Public Health and Tropical Medicine, Tulane University, New Orleans, USA; 3 Louisiana State University Health Sciences Center Comprehensive Alcohol and HIV Research Center (CARC), New Orleans, LA USA; 4 Louisiana State University Health Sciences Center School of Public Health, New Orleans, LA USA; 5 Louisiana State University Health Sciences Center School of Medicine, New Orleans, LA USA; 6 Department of Epidemiology, Celia Scott Weatherhead School of Public Health and Tropical Medicine, Tulane University, New Orleans, USA

**Keywords:** Life course, adverse childhood experiences, urban stress, latent class analysis, continuum of care

## Abstract

People with HIV (PWH) have often experienced chronic stressors across their lifespan, including adverse childhood experiences (ACES), lifetime economic hardship (LEH), and concurrent stressors associated with living in urban areas (urban stress). Prolonged exposure to stressors might result in differential coping patterns among PWH that can impact care trajectories. We utilized a life course-informed approach to examine chronic stressors as antecedents of latent coping strategies among PWH in care. High-risk alcohol use and non-adherence to anti-retroviral therapy (ART) were examined as consequences of latent coping strategies. Data were utilized from the baseline and interim follow-up visit of the New Orleans Alcohol Use in HIV (NOAH) study. Three latent classes of coping strategies were identified: avoidance coping (31%), low-frequency coping (43%), and problem-solving coping (25%). Exposure to ACES was associated with greater use of avoidance versus low-frequency coping class at wave II. Urban stress was associated with greater use of avoidance coping compared to problem-solving or low-frequency coping classes at wave II. LEH was associated with greater use of low-frequency coping at wave II. Those utilizing low-frequency coping had a two-fold increase in ART non-adherence compared to problem-solving coping. PWH utilizing avoidance and low-frequency coping had a nearly two-fold increase in high-risk alcohol use versus problem-solving coping. These findings reveal important coping classifications that are linked to stressors across the life course of PWH. An understanding of coping styles and stressors may aid in improving the continuum of care among PWH by reducing alcohol use and improving medication adherence.

## Introduction

Coping and stress theories posit that individuals develop coping strategies in response to chronic stressors which might mitigate or perpetuate the impact of stressors on health and well-being [1, 2]. Adverse childhood experiences (ACES) are a potent chronic stressor and adaptive coping such as problem-solving [2] and maladaptive coping such as avoidance have been associated with ACES [3]. Individuals exposed to ACES might not develop effective problem-solving skills necessary for stress mitigation [2]. From an early age, research suggests that ACES may lead to cognitive schemas that reinforce a belief in the inability to effect change [4]. In support of this, in a study examining childhood coping with chronic abuse, maltreated children relied heavily on denial and employed strategies to avoid the abusive caregiver [4]. Although such strategies might be functional in childhood to escape chronic abuse [4] they have been proven to be less adaptive in adulthood. For example, avoidance-focused coping was seen to mediate the relationship between ACES and psychopathologies among adults [3].

While longitudinal studies provide reasonable evidence that early life stressors are antecedent to differential coping strategies [3, 5], concurrent stressors have rarely been examined as predictors of coping [6]. Urban life stress is a concurrent stress associated with living in cities and encompasses economic instability, racism, and neighborhood contexts [7]. Cross-sectional studies suggest that neighborhood contexts such as perceived crime are associated with avoidance coping strategies like substance use and venting [8]. Similarly, individuals exposed to racism may be more likely to employ avoidance coping strategies such as denial to minimize the emotional distress associated with experiencing racism [9]. Adding to these complexities is lifetime economic hardship (LEH), a pervasive factor closely related to early life and concurrent stress that might profoundly influence coping mechanisms. LEH is characterized by prolonged financial instability that can multiply the impact of ACES and urban stress and limit an individual’s capacity to cope adaptively. The economic burden can not only limit access to healthcare but also increase reliance on maladaptive coping strategies such as substance use and avoidance [10]. To our knowledge, no studies have examined the combined impact of early life, concurrent stressors, and lifetime economic hardship on coping strategies among PWH.

Chronic stressors are pervasive among PWH, with more than one-third of PWH having experienced sexual abuse before the age of 18 [11], and nearly one in six having experienced urban life stressors like racism [12] in some samples. Understanding the differential use of coping in response to stress might help identify stress mitigation strategies and improve the continuum of care among PWH. A few studies have examined the impact of coping strategies on medication adherence among PWH [13–15]. For example, maladaptive coping strategies such as avoidance and denial were associated with missed doses among women living with HIV [14]. PWH utilizing adaptive coping strategies such as positive thinking and seeking information were more likely to be adherent to medications [13]. Apart from medication adherence, positive coping might also reduce high-risk alcohol use among PWH, improving their adherence to treatment and overall wellbeing [16, 17]. For example, positive coping strategies such as action and coping through religion were seen to reduce alcohol use among PWH [16, 17].

While studies have examined coping strategies and their impact on health outcomes among PWH, most utilize approaches that fail to recognize coexisting coping strategies [16, 18, 19]. For example, only one study examined coexisting latent profiles of coping strategies among PWH [20]. The latent class approach is promising in examining coexisting coping strategies among PWH and predicting the impact of chronic stressors. Such an approach may be instrumental in identifying individuals at higher risk for maladaptive coping strategies and in helping to develop individually tailored but context-specific interventions.

Hence, the objective of this study was to examine the latent classes of coping strategies among a sample of in-care PWH, explore the roles of ACES, urban life stress, and lifetime economic hardship as antecedents of the latent classes, and examine the association of the latent classes with alcohol use and ART adherence which can impact the continuum of care of PWH. The hypotheses of the study were threefold. Firstly, latent classes of coping strategies will be identified and differentiated according to adaptive or maladaptive coping typologies among PWH. Secondly, chronic stressors will be predictive of latent class profiles and will result in greater use of maladaptive coping strategies by PWH. Thirdly, latent classes of coping will be associated with alcohol use and ART adherence among PWH. Specifically, maladaptive coping will be positively associated with poor medication adherence and high-risk alcohol use among PWH.

## Methodology

### Study Population

The current study utilized data from an ongoing longitudinal study called the New Orleans Alcohol Use (NOAH) Study conducted by the Comprehensive Alcohol-HIV/ AIDS Research Center (CARC) at the Louisiana State Health Sciences Center in New Orleans, Louisiana [21]. The cohort included adult PWH, 18 years and above engaged in care at a local clinic. Other inclusion criteria were an absence of pregnancy, acute illness, or intoxication at baseline visit. The recruitment process is detailed in a previous publication [21]. We utilized the data from the baseline and interim follow-up visits. Study staff recruited 365 PWH in Wave I of the study conducted between 2015 and 2017, which was considered the baseline. Of the 365 PWH enrolled in Wave I, 298 were retained at interim follow-up visits in Wave II of the study conducted between 2018 and 2019. Louisiana State Health Sciences Center and Celia Scott Weatherhead School of Public Health and Tropical Medicine, Tulane University in New Orleans provided the ethical approvals for the study.

### Measures

#### Antecedents of Coping

##### Adverse Childhood Experiences

Adverse childhood experiences (ACES) were measured by a 10-item retrospective inventory of experiences of sexual, physical abuse, and neglect before 18 years of age [22]. We created the total ACES score by summing the affirmative response. A dichotomous variable was created by utilizing previous reports of a threshold of 4 or more ACES indicating a high exposure to early adversity [23–25]. ACES was assessed during the interim follow-up visit at Wave II of the study. Previous studies have utilized the 10-item ACES inventory in this sample [26, 27] and in other studies among PWH [25, 28].

##### Urban Life Stress

Urban life stress was measured by utilizing a 21-item inventory of perception of stress in response to adverse experiences associated with living in an urban environment, also called the urban life stressor scale (ULSS) [7]. Stress perception is measured on a Likert scale of 1 to 5, where 1 corresponds to ‘no stress at all’ and 5 to ‘extremely stressful’. We created a score by summing the responses. Urban life stress was assessed during baseline at Wave I of the study. ULSS has been utilized in another study in this sample [29].

##### Lifetime Economic Hardship

Lifetime economic hardship (LEH) was measured by utilizing a six-item inventory of financial difficulties, such as not being able to pay the rent or mortgage, at four time points across the life span: as a child (< 12 years), as an adolescent (12–17 years), as an adult (18 + years) before HIV diagnosis, and since HIV diagnosis. All were answered Yes/ No. We created a score by summing the affirmative responses. LEH was recorded during baseline at Wave I of the study. This scale has been adapted from existing scales on financial strain among individuals with and without HIV [30, 31].

#### Coping

Coping was measured by utilizing a 28-item inventory to measure individual responses to stressful events [32]. The inventory contains 14 subscales corresponding to problem-focused, emotion-focused, and avoidance coping [32]. Problem-focused coping contains four subscales of two items each, which are active coping, use of informational support, positive reframing, and planning [32]. Emotion-focused coping contains 6 subscales of two items each, which are seeking emotional support, venting, humor, acceptance, religion, and self-blame [32]. Avoidance coping contains four subscales of two items each, which are self-distraction, denial, substance use, and behavioral disengagement [32]. The responses are recorded on a Likert scale of 1 to 4, where 1 corresponds to ‘not utilizing the strategy at all’ and 4 to ‘utilizing the strategy a lot’. We created scores for each subscale and dichotomized each subscale using the 75th percentiles. Coping was assessed at the interim follow-up visit. Coping scale has been utilized in other studies in the context of HIV [33, 34].

#### Consequences of Coping

##### High-risk alcohol use

High-risk alcohol use was categorized by utilizing the timeline follow-back (TLFB) questionnaire. Participants recalled the exposure to alcohol over the past 30 days, including the number of drinks and types they consumed. National Institute on Alcohol Abuse and Alcoholism (NIAAA) criteria were utilized to categorize High-risk alcohol use [35]. High-risk alcohol use was defined as more than 3 drinks for women or more than 4 drinks for men on any single day and more than 7 drinks per week for women or 14 drinks per week for men. TLFB was assessed at the interim follow-up visit.

##### Adherence

Adherence to HIV medication in the past three months was categorically defined as adherent for PWH who took 100% of their doses and non-adherent for those who missed any dose [29]. Adherence was determined by the participants’ self-reports of the percentage of medications they took over the last three months. Responses were recorded on a scale of 1–4, where ‘1’ corresponds to < 50% of the dosage taken and ‘4’ corresponds to 100% dosage taken. Adherence was assessed at the baseline.

#### Covariates

Covariates included participant demographics such as age, reported sex (male vs. female), sexuality (bisexual and lesbian/ gay indicators), race (black vs. others), and educational attainment (below primary as low and above primary as high), which were collected at baseline.

### Statistical Analysis

Data were managed in SAS version 9.4. A preliminary descriptive analysis was implemented in SAS to describe the sociodemographic characteristics of the study population, the prevalence of ACES, coping strategies, mean urban life stress scores, and LEH. Latent class analysis (LCA) of coping strategies and the association of latent classes by ACES and urban life stress were conducted in MPLUS version 8. Class membership was ascertained by utilizing the goodness of fit statistics like Akaike information criteria (AIC), Bayesian Information Criteria (BIC), Sample size adjusted Bayesian Information Criteria (SSBIC), as well as entropy along with interpretability of the latent classes. Lower AIC, BIC, and SSBIC indicate a model with a better fit, while a higher entropy close to 1 indicates well-differentiated classes. Additional statistical tests comparing k and k + 1 classes were implemented [36]. These are the Lo-Mendell-Rubin likelihood test (LMR) and Bootstrap Likelihood Ratio test (BLRT) [36]. The default LCA estimator of maximum likelihood was utilized.

The auxiliary function in MPLUS was utilized to estimate latent class membership by ACES, urban life stress, and LEH while adjusting for age, education, sex, sexuality, and race as covariates [37]. Models estimated using the Vermunt-three-step approach might be superior to the traditional or naive three-step approach [38]. The traditional three-step approach involves the estimation of the latent classes in step one, assigning classes based on posterior probabilities in step two, and predicting the membership of classes based on the posterior probabilities, which might result in a classification error [38]. The three-step approach estimates the latent classes, where the first two steps are the same as the traditional approach. They differ in the last step, where a nominal latent class indicator is assigned to predict the membership of latent classes. By doing so, the coefficients of the multinomial regression of the assigned class are based on the true class, thus minimizing the classification error noted in the naive three-step approach [38, 39]. Logistic regression was conducted in SAS to examine the association between latent class typologies and distal variables of high-risk alcohol use and adherence to antiretroviral therapy (ART). ACES, urban life stress, and LEH, were also covariates in the logistic regression models as they might confound the relationship between the coping typologies and the outcomes.

## Results

Seven participants were missing data on coping variables, and three were missing sexuality variables, which were excluded from the analysis, leaving an analytical total of 288. The mean age of the sample was 49 years (Table 1). Most of the sample self-identified as Black/ African American (82.6%), one-third as female (31.6%), approximately 40% had less than a high school education, 12.9% identified as bisexual, and 23.6% identified as lesbian or gay. High-risk alcohol use and non-adherence were reported by 41.0% and 58.3% of the study population, respectively. Four or more ACES were reported by 42.0% of the study population. Among the problem-solving coping, the highest percentage was reported for active coping (37.8%), followed by planning (28.8%), seeking instrumental support (28.5%), and positive reframing (27.8%). Among emotional coping strategies, the sample reported the highest percentage of acceptance (62.2%), followed by religious coping (44.8%), self-blame (33.3%), venting (33.3%), and humor (29.9%). Among the avoidance coping strategies, the highest percentage was reported for substance use (33.7%), followed by disengagement (29.9%), self-distraction (28.5%), and denial (28.5%). The mean urban life stress score was 46.05 (Standard deviation (SD) = 18.22). The mean LEH score was 6.12 (SD = 5.46).

**Table 1:** Characteristics of the study population (*N* = 288)

	*N* %	Mean (SD)	Median (75th /25th percentile)
Age		49.07 (10.05)	51.00 (56.00/42.00)
Reported sex			
Female	91 (31.6)		
Male	197 (68.4)		
Self-reported race			
Black/ African American	238 (82.6)		
Other	50 (17.4)		
Education			
Less than high school	115 (39.9)		
High school graduate and higher	173 (60.1)		
Sexuality			
Bisexual	37 (12.9)		
Lesbian or Gay	68 (23.6)		
Alcohol use			
High risk	118 (41.0)		
Low risk	170 (59.0)		
HIV medication			
No missed pill	120 (41.7)		
Missed at least one pill	168 (58.3)		
High Adverse Childhood Experiences (ACE) threshold			
< 4	167 (58.0)		
>=4	121 (42.0)		
Coping			
Active coping	109 (37.8)		
Instrumental support	82 (28.5)		
Positive reframing	80 (27.8)		
Planning	83 (28.8)		
Emotional support	77 (26.7)		
Venting	96 (33.3)		
Humor	86 (29.9)		
Acceptance	179 (62.2)		
Religion	129 (44.8)		
Self-blame	96 (33.3)		
Self-distraction	82 (28.5)		
Denial	82 (28.5)		
Substance use coping	97 (33.7)		
Disengagement	86 (29.9)		
Urban life stress		46.05 (18.22)	43.00 (56.00/31.50)
Lifetime economic hardship (LEH)		6.12 (5.46)	5.00(8.00/2.00)

Model fit indices are provided in Table 2. Following the recommendations of Nylund et al., to utilize BIC and LMR coupled with theoretical interpretability as a guide to latent class enumeration, a three-class model was selected [40]. A non-significant value of LMR is a good indication to stop the addition of classes [40], which was seen in the three-class model (*p* = 0.07). Among the information criteria, BIC (4714.70) was the lowest for the three-class model, SSBIC for the five-class model (4526.30), and AIC for the five-class model (4489.91). The entropy did not improve for coping across the five classes examined. The AIC is not a good indicator for class enumeration and frequently overestimates the classes. SSBIC may outperform BIC in studies with small sample sizes (less than 200) [40], which is not the case in this study. Since BIC has outperformed other information criteria in selecting latent classes [40, 41], we selected the three-class model with the lowest BIC. The BLRT did not improve upon class addition for all the five classes utilized to build the model. We relied on BIC for model interpretation because we note inconsistencies in the LMR and BLRT probability values. Also, the three-class model generated theoretically plausible results.

**Table 2:** Fit statistics for latent classes of coping strategies

	Entropy	AIC	BIC	SSBIC	LMR-LRT	BLRT
2 CLASS	0.80	4647.27	4753.50	4661.53	0.00	0.00
3 CLASS ^a^	0.79	4553.52	4714.70	4575.17	0.07	0.00
4 CLASS	0.79	4552.56	4722.54	4535.19	0.17	0.00
5 CLASS	0.80	4489.91	4760.97	4526.30	0.31	0.01

AIC- Akaike Information Criteria; BIC- Bayesian Information Criteria; SSABIC- Sample size adjusted BIC; LMR-LRT- p value for Lo-Mendell-Rubin Likelihood Ratio Test; BLRT- p value for Bootstrap Likelihood Ratio test

^a^ selected as the final model

**Fig. 1:**
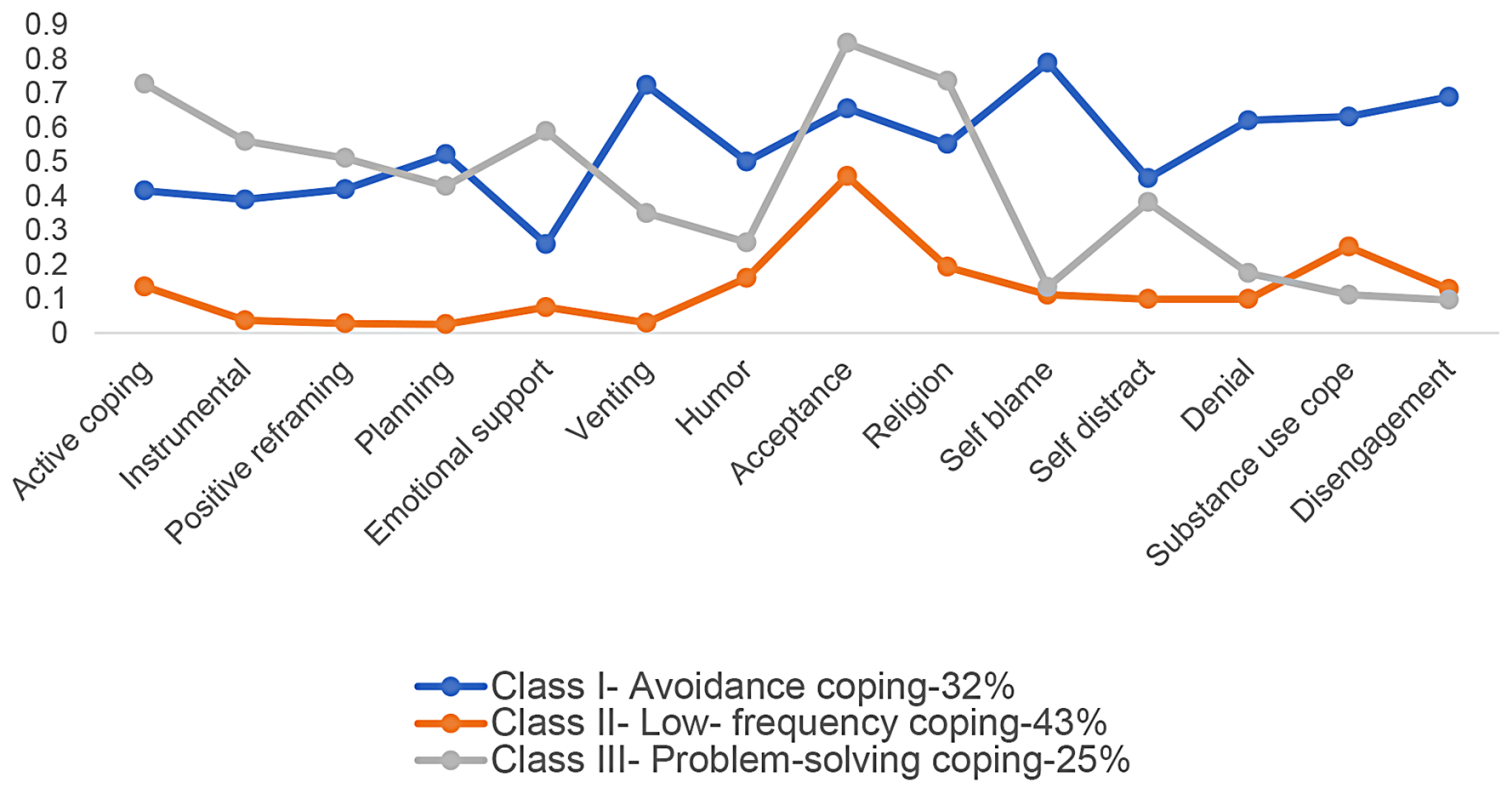
Conditional probability plots of coping strategies for the three latent classes

PWH in Class I or the avoidance coping class (31.6%) had a high probability of utilizing avoidance coping strategies, namely self-distraction (45.2%), denial (62.1%), substance use (63.2%), and disengagement (68.9%) (Table 3; Fig. 1). PWH in Class I also had a high probability of dysfunctional emotional coping strategies such as self-blame (79.0%) and venting (72.4%). PWH in Class II of low-frequency coping (43.4%) had a low probability of all coping strategies. PWH in Class III or problem-solving coping (25.0%) had a high probability of problem-solving coping strategies such as active coping (72.8%), seeking instrumental support (56.1%), and positive reframing (51.1%). They also employed higher functional emotion coping strategies such as acceptance (84.7%) and religion-focused coping (73.7%).

**Table 3:** Conditional probability of coping strategies for the three latent classes

	Class I: avoidance- coping (31.6%, *n* = 91)	Class II: low- frequency coping (43.4%, *n* = 125)	Class III: problem- solving coping (25.0%, *n* = 72)
Active coping	41.6	13.6	72.8
Instrumental support	39.0	3.7	56.1
Positive reframing	42.0	2.8	51.1
Planning	52.2	2.6	42.9
Emotional support	26.0	7.6	58.9
Venting	72.4	3.0	35.0
Humor	50.1	16.1	26.5
Acceptance	65.6	45.8	84.7
Religion	55.2	19.3	73.7
Self-blame	79.0	11.2	13.5
Self-distraction	45.2	1.0	38.2
Denial	62.1	1.0	17.5
Substance use coping	63.2	25.2	11.2
Disengagement	68.9	12.8	9.7

ACES, urban life stress, and LEH predicted coping strategies at Wave II of the study (Table 4). PWH exposed to four or more ACES were more than twice as likely (OR = 2.87, CI = 1.34–6.16) to employ avoidance coping strategies compared to low-frequency coping after controlling for reported sex, sexuality, age, race, education, urban life stress, and lifetime economic hardship. Low-frequency coping was included as the referent class, as previous research suggests that poor coping responses might be seen in the absence of a stressful stimulus in the social environment [42]. Alternately, low coping in the presence of a stressful stimulus might indicate giving up the will to cope, which might be the most deleterious to health [43]. While PWH with high ACES thresholds were also more likely to be in the avoidance coping class compared to the problem-solving coping class, the results were non-significant (OR = 1.86, CI = 0.77–4.45). Problem-solving coping was utilized as a referent class, as previous research suggests it is more adaptive than avoidance coping [5]. Each unit increase in urban life stress at Wave I of the study was associated with a 3% increase in being in the avoidance coping class compared to low frequency coping class (OR = 1.03, CI = 1.01–1.06) and of being in the avoidance coping class compared to problem-solving coping class (OR = 1.03, CI = 1.01–1.06) at the Wave II of the study, after controlling for covariates, ACES, and LEH. A one-unit increase in LEH was also associated with an 8% increase in being in the low-frequency coping class (OR = 1.08, CI = 1.00-1.17) compared to the problem-solving coping class after controlling for covariates, ACES, and urban life stress.

**Table 4:** Factors associated with coping strategies latent class membership

Odds ratios (95% confidence interval)	Class I- Avoidance- coping	Class II – Low-frequency coping	Class III- Problem- solving Coping
***Parametrization with reference Class 2***			
ACES < 4	Ref	-	Ref
>=4	2.87 (1.34–6.16) **	-	1.55 (0.69–3.46)
Urban life stress	1.03 (1.01–1.06) **	-	1.00 (0.98–1.03)
Lifetime economic hardship	0.96 (0.90–1.02)	-	0.92 (0.85–0.99) **
Reported sex Female	Ref	-	Ref
Male	0.84 (0.37–1.88)	-	1.06 (0.45–2.42)
Sexuality			
Bisexual	0.49 (0.18–1.37)		0.34 (0.10–1.24)
Lesbian or gay	0.54 (0.19–1.51)		
Age	0.94 (0.91–0.98)	-	0.96 (0.93-1.00)
Education High school graduate and higher	Ref	-	Ref
Less than high school	1.14 (0.56–2.34)	-	0.98 (0.46–2.05)
Race			
Others	Ref	-	Ref
Black	1.94 (0.69–5.45)	-	1.12 (0.47–2.95)
***Parametrization with reference to Class 3***			
ACES			
< 4	Ref	Ref	
>=4	1.86 (0.77–4.45)	0.65 (0.46–2.74)	-
Urban stress	1.03 (1.01–1.06) **	1.00 (0.97–1.02)	-
Lifetime economic hardship	1.04 (0.95–1.13)	1.08 (1.00-1.17) **	-
Reported sex			
Female	Ref	Ref	
Male	0.79 (0.32–1.97)	1.01 (0.44–2.29)	-
Sexuality			
Heterosexual	Ref	Ref	
Bisexual	1.43 (0.36–5.59)	2.91 (0.80-10.53)	-
Lesbian or gay	0.97 (0.30–3.20)	1.79 (0.69–4.68)	-
Age	0.98 (0.94–1.02)	1.04 (1.00-1.08)	-
Education			
High school graduate and higher	Ref	Ref	
Less than high school	1.17 (0.51–2.70)	1.03 (0.49–2.15)	-
Race			
Others	Ref	Ref	-
Black	1.65 (0.51–5.33)	0.85 (0.34–2.13)	

** *p* < 0.05

Latent classes of coping were associated with high-risk alcohol use and ART non-adherence among PWH after controlling for gender, sexuality, age, education, race, ACES, urban life stress, and LEH. Low-frequency coping was associated with a two-fold (OR = 2.31, CI = 1.22–4.38) increase in high-risk alcohol use and a nearly two-fold (OR = 1.90, CI = 1.01–3.56) increase in non-adherence compared to problem-solving coping. Avoidance coping was also associated with a nearly two-fold increase in high-risk alcohol use compared to problem-solving coping (OR = 1.93, CI = 0.96–3.87). Among the life course stressors, only LEH was associated with ART non-adherence. A unit increase in LEH was associated with a 7% increase in ART non-adherence (OR = 1.07, CI = 1.01–1.13).

**Table 5:** Association between coping strategies latent classes and high-risk alcohol use and non-adherence among people with HIV

Adjusted odds ratio (95% CI)	High-risk alcohol use vs. Low risk alcohol use	Non-adherence vs. adherence
Coping latent classes		
Class I- Avoidance coping	1.93 (0.96–3.87) *	1.75 (0.89–3.45)
Class II-Low-frequency coping	2.31 (1.22–4.38) **	1.90 (1.01–3.56) **
Class III-Problem solving coping	Ref	Ref
ACES		
< 4	Ref	Ref
>=4	1.27 (0.74–2.20)	0.74 (0.42–1.31)
Urban life stress	0.98 (0.97-1.00)	1.01 (1.00-1.03)
LEH	1.01 (0.96–1.05)	1.07 (1.01–1.13) **
Reported sex		
Female	Ref	Ref
Male	1.53 (0.85–2.74)	0.92 (0.50–1.68)
Education		
High school graduate and higher	Ref	Ref
Less than high school	1.44 (0.86–2.40)	0.98 (0.58–1.68)
Age	0.98 (0.96–1.01)	0.99 (0.96–1.01)
Sexuality		
Bisexual	1.20 (0.56–2.54)	0.38 (0.170–0.87) **
Lesbian or gay	0.96 (0.49–1.87)	0.81 (0.41–1.62)
Race		
Others	Ref	Ref
Black	1.43 (0.72–2.85)	2.80 (1.39–5.62) **

** *p* < 0.05; * *p* < 0.10

## Discussion

The study examined clusters or classes of coping strategies among a cohort of in care PWH and the impact of early life, lifetime, and concurrent chronic stressors on latent class membership. The findings support our hypothesis that latent classes of coping strategies would be identified and would be differentiated according to adaptive and maladaptive coping. We identified three latent classes of coping strategies employed by PWH. PWH in the avoidance coping class (Class I; 31.6%) constituted a high percentage of maladaptive coping strategies. PWH in the problem-solving coping class (Class III; 25.0%) constituted a high percentage of adaptive coping strategies, and PWH in the low frequency coping class (Class II; 43.4%) constituted a low percentage of all strategies.

Similar to the current study, Nylund-Gibson et al. reported three latent classes—externalizing, adaptive coping, and no coping [44]—corresponding to avoidance, problem-solving, and low-frequency coping classes in the current study. However, Yuan and colleagues identified three parallel coping profiles without differentiating adaptive and maladaptive coping strategies. These were high, medium, and low coping strategies [45]. Another study identified three latent classes but lacked a low coping class [46]—two were maladaptive: avoidant copers, and disengaged copers, and the third was adaptive: engaged copers [46]. Variations in the target populations might account for the differences in the latent class categorization in these studies. While the current study focused on PWH, Yuan et al. focused on adolescents, and Kavcic et al. on adults affected by the COVID-19 lockdown. However, none of the studies differentiated an emotion-focused coping class. This finding is consistent with the lack of consensus on attributing emotion-focused coping as either maladaptive or adaptive [47, 48].

While emotion-focused coping, such as seeking social support and acceptance, is considered adaptive [49–52], differences can be noted in the classification of religion [6, 53], venting [47, 48], and self-blame [54] as adaptive or maladaptive. Religion has been considered adaptive among people exposed to prolonged suffering due to an illness [55] or violence [53]. However, others contest it to be more maladaptive [55, 56]. Greater focus on religious beliefs, such as the disease as a punishment from God, has been associated with extreme emotional reactivity [55] and preoccupation with ritual that diverts focus from removing the stressor [56]. In our study, coping through religion was seen to be higher in the problem-solving coping class which suggests it might be more adaptive for this study population.

Similarly, venting and self-blame have been associated with adverse impacts such as psychological distress among adults experiencing stressful situations such as caring for mentally ill patients [57]. A study also found a positive association between venting and poor quality of life among PWH [58]. Venting has been posited to be maladaptive as it may amplify the negative emotional state and is associated with greater rumination about the stressor [47]. In contrast, others consider venting adaptive among people with low perceived support [48]. Similar contextual differences were seen in the categorization of self-blame, such that behavior-focused self-blame was regarded as adaptive compared to individual-focused self-blame [54]. Behavior-focused self-blame might be more adaptive as individuals appraise the negative consequences of their actions and recognize their behavior as modifiable [54].

While the categorization of venting and self-blame as adaptive and maladaptive might vary according to social contexts, PWH in the avoidance coping class had a higher probability of both negative health behaviors in our study. This categorization is also consistent with the premise that chronic stressors lead to the development of maladaptive coping strategies. Our data show that PWH exposed to chronic urban stress at baseline were more likely to utilize avoidance coping compared to problem-solving coping. Similarly, Le and colleagues found that racism among a cohort of Asian American college students was associated with greater substance use coping over time [6].

We found partial support for the hypothesis that chronic stressors lead to increased maladaptive vs. adaptive coping strategies among PWH. While we identified ACES as an antecedent to coping strategies, we did not find it to be associated with greater use of avoidance coping compared to problem-solving coping. Exposure to ACES resulted in a nearly two-fold increase in avoidance coping class compared to problem-solving. However, these differences did not reach statistical significance. These findings contrast with those of a longitudinal study that found greater use of avoidance coping compared to problem-solving coping among participants reporting ACES [3]. Interestingly, we found ACES to be associated with greater use of avoidance coping compared to low-frequency coping. These findings are in line with the stress theories, which posit that a negative coping response would be observed in response to a stressful stimulus [42]. However, an absence of a coping response can also be harmful [43].

The harmful nature of low-frequency coping and avoidance coping strategies was better ascertained by examining its association with high-risk alcohol use and ART nonadherence, which has implications for the continuum of care among PWH (Hypothesis III). Both avoidance and low-frequency coping were associated with adverse health outcomes, suggesting both strategies to be maladaptive among PWH. PWH in the avoidance coping class were two-times more likely to be high-risk alcohol users compared to those in the problem-solving coping class. Findings are consistent with another study that found increased missed doses among women employing avoidance coping strategies [14].

The low-frequency coping class was associated with a two-fold increase in both high-risk alcohol use and non-adherence among PWH compared to problem-solving coping. These findings are supported by those of Ali et al., that suggest low coping to be most detrimental to health, as they found low coping mechanisms or giving up the will to cope to be associated with greater anxiety and stress levels among health care providers. In contrast, a study among PWH found subjective well-being to be better among PWH with low coping profiles compared to mixed or high-intensity coping profiles [46]. Because studies are inconsistent in classifying low coping behaviors as adaptive or maladaptive, further research is required to understand low coping behaviors among PWH.

Our data suggest that ACES and urban life stress at wave I resulted in greater use of avoidance coping strategies at wave II among PWH, and the use of avoidance coping was associated with increased high-risk alcohol use at wave II. Urban life stress was also associated with greater use of low-frequency coping, and low-frequency coping was associated with ART non-adherence. LEH was also associated with increased low-frequency coping and with ART nonadherence, indicating a pathway to ART nonadherence. While our research did not indicate any relationship between ACES/ urban life stress and high-risk alcohol use and ART non-adherence, earlier research among the same cohort of PWH found a positive association between chronic stressors and alcohol use [26, 29] among PWH, which suggests a pathway to alcohol use. Future research is required to examine the mediating/ moderating roles of latent coping classes between life course stressors (ACES, urban stressors, LEH) and the continuum of care among PWH.

The strengths of the study are the utilization of a life course approach to examine early life, concurrent, and lifetime stressors as predictors of latent classes of coping strategies employed by PWH. Our study is the first to examine ACES, urban life stress, and LEH as antecedents of latent classes of coping strategies among PWH and to examine the roles of urban life stress encompassing racism, economic stress, and neighborhood stressors on coping. We have leveraged a longitudinal study design to maintain the temporality of the variables investigated. Urban life stress was recorded at baseline, and coping was recorded at the interim follow-up visit of the study. While ACES were recorded at the interim visit, the temporality is maintained as it is a retrospective measure generating information on stressors in the first 12 years of age. Another consideration is the limitation of the retrospective nature of ACES measurement, which might result in recall bias and under-reporting of abuse [59]. We were also unable to establish the temporality of the adherence measure, which was recorded at baseline. Another limitation is the study’s generalizability. The study included in-care low-income PWH in the South, and findings might not be generalizable to PWH in other areas and those not in care.

Findings suggest that health care providers should screen PWH for exposure to ACES, urban life stressors such as racism and neighborhood crime, and LEH to provide context-specific care. Stress mitigation therapies should target the development of problem-focused coping strategies among PWH to improve health outcomes such as alcohol use and medication adherence. Policymakers should work to strengthen the social welfare systems to prevent early childhood adversity, ensure a healthy home environment for children to thrive, and provide safe neighborhood spaces by reducing vacant and abandoned property and neighborhood crime. Income-strengthening measures to minimize the impact of economic headship might also improve coping behaviors among PWH. Future studies should focus on understanding the pathways between chronic stressors, coping, and physiological and psychological health outcomes among PWH. The development of individually tailored interventions through latent class approaches may aid in improving coping strategies employed by PWH. Incorporating context-specific strategies to mitigate lifetime exposure to stressors such as ACES, LEH, and urban life stress might enhance the impact of coping interventions.
