# Correction: Life Course Stressors, Latent Coping Strategies, Alcohol Use, and Adherence among People with HIV

**DOI:** 10.1007/s10461-024-04570-1

**Published:** 2024-12-18

**Authors:** Amrita Gill, Gretchen Clum, Patricia Molina, David Welsh, Tekeda Ferguson, Katherine P. Theall

**Affiliations:** 1 Department of Psychiatry and Human Behavior, Warren Alpert Medical School of Brown University, Box G-BH, 02912 Providence, RI USA; 2 Department of Social, Behavioral and Population Sciences, Celia Scott Weatherhead School of Public Health and Tropical Medicine, Tulane University, New Orleans, USA; 3 Louisiana State University Health Sciences Center Comprehensive Alcohol and HIV Research Center (CARC), New Orleans, LA USA; 4 Louisiana State University Health Sciences Center School of Public Health, New Orleans, LA USA; 5 Louisiana State University Health Sciences Center School of Medicine, New Orleans, LA USA; 6 Department of Epidemiology, School of Public Health and Tropical Medicine, Celia Scott Weatherhead, Tulane University, New Orleans, USA

In this article the title was incorrectly given as “Life Course Stressors, Latent Coping Strategies, Alcohol Use, and Latent Coping Strategies Among People with HIV” but should have been “Life Course Stressors, Latent Coping Strategies, Alcohol Use, and Adherence among People with HIV”.

The original article has been corrected.

